# Supplementary material for: Nutrient Intake during Pregnancy and Adherence to Dietary Recommendations: The Mediterranean PHIME Cohort
Source: Nutrients. 2021 Apr 24;13(5):1434. doi: 10.3390/nu13051434 (PMC8146946; doi:10.3390/nu13051434)

Figure S1 – Flow-chart of the Mediterranean PHIME cohort

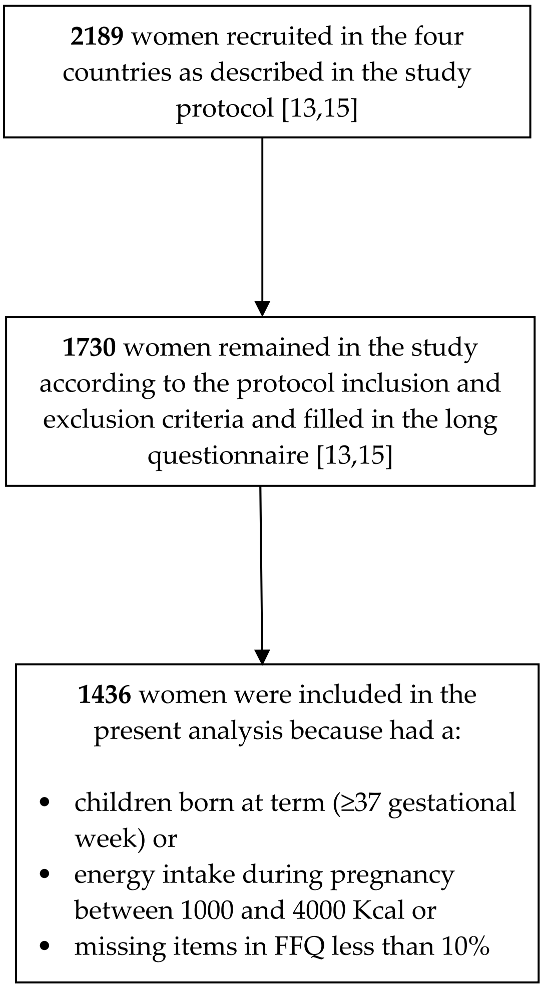

Table S1 – Food groups and food item constituents as listed in the food-frequency questionnaire.

Food groups	Food item
Fruit	Oranges; mandarin oranges, tangerines, other citrus fruits; grapefruit; lemons: e.g., a vegetable, in tea or other; fresh squeezed citrus fruits: orange, grapefruit, lemon, etc. Apples; pears; watermelon; banana; strawberries; cherries; kiwi; melon; peaches, nectarines, apricots; plums; figs, grapes, pineapple, other exotic fruits; ½ cooked fruits (sugared fruits).
Vegetables	Spinach, swiss chard, chicory, dandelion greens; artichokes, cardoon; cauliflower; cabbage, savoy cabbage, broccoli, turnip greens; mushrooms; aubergines; peppers (red, yellow, green); zucchini; pickled vegetables (pickles, spring onions, mixed etc); green or red salad; mixed salad with carrots, tomatoes, cucumbers; green beans; tomatoes.
Potatoes	potatoes: mashed or boiled; potatoes: fried or roasted; potato chips, salted snacks.
Cereals based products	White bread; whole wheat bread; crackers, breadsticks, melba toast; whole wheat crackers (and breadstick and melba toast); breakfast cereals; polenta; pasta or rice with oil or butter, risotto; pasta or rice with tomato sauce; pasta or rice with vegetables; lasagne, tortellini, moussaka with vegetables; ½ soups or other first courses with cereals (barley, spelt, pearl millet); ½ pizza; ½ pasta or rice with meat sauce; ½ lasagne, tortellini, moussaka with meat sauce.
Milk and dairy products	Grated cheese, on first course pasta and soups; fresh cheese (ricotta, mozzarella, kasseri) as a main course; feta as a main course; other cheeses as a main course; cheese as a last course or snack; cow's milk, whole; cow's milk, skim or low fat; goat's milk; plain or fruit yogurt, whole; plain or fruit yogurt, low fat; ½ milk in coffee or tea; ½ cappuccino or coffee latte; ½ pizza.
Meat	Pork meat, prepared in any manner; raw ham, smoked ham, dried beef; cooked ham; salami, mortadella, bacon, etc; sausage, frankfurters, cotechino (Cevapcici, musetto, etc); lean meats (roast-beef, steak, etc): veal, beef or horse; meat stew (goulash), pot roast, meatballs, cutlet, roast: veal, beef or horse; chicken or turkey with little added seasoning or boiled; chicken or turkey roasted, fried or stewed; rabbit, prepared in any manner; goat meat, prepared in any manner; lamb meat, prepared in any manner; liver meats, any animal; other organ meats: heart, brain, kidneys, thymus; wild game meats (hare, pheasant, venison, etc); ½ pasta or rice with meat sauce; ½ lasagne, tortellini, moussaka with meat sauce.
Fish and seafood	Fish: boiled, grilled, baked; crustaceans (lobster, crab, shrimp) boiled, grilled, baked; molluscs (squid, cuttlefish, clam) boiled, grilled, baked; fried fish; fried crustaceans; fried molluscs; tuna, mackerel, sardines in oil.
Eggs	Egg: hard boiled, soft boiled, poached or raw; egg: fried, scrambled, omelette
Pulses	Beans, fresh and dry; peas, fresh and dry; other legumes (chickpeas, lentils, fava beans); soy milk; soy and soy products (beans, sprouts, tofu, etc); ½ vegetables soup, pasta with beans.
Nuts and dried fruit	Dried fruits: walnuts, hazelnuts, almonds, etc; raisins, dried prunes, dried figs, etc; green or black olives (natural or salted).
Soup	Broth - also with pasta or rice; ½ vegetables soup, pasta with beans; ½ soups or other first courses with cereals (barley, spelt, pearl millet).
Sweet and dessert	Powdered cocoa; cookies (all types except whole wheat); whole wheat cookies; croissants, doughnuts, commercially prepared cakes, sponge cakes or other leavened cakes, without cream, etc; pastries with cream, cream filled doughnuts; fruit or jam tart, pastries with fruit; puddings, cream caramel, spoon desserts; chocolate, chocolate candy; chocolate bars such as mars, kit-kat, bounty; candies "normal"; ice cream.
Sugar	Sugar added to drinks or other foods; candies "light" (without sugar); chewing gum "normal"; chewing gum "light" (without sugar); jam or honey; pop corn; coca

	cola, Pepsi cola, chinotto; other carbonated soft drinks without alcohol; light drinks (without sugar); commercial fruit juices; ½ cooked fruits (sugared fruits); artificial sweeteners, saccharin, aspartame.
Coffee, tea and chocolate	Tea; herbal tea, decaffeinated tea; coffee; decaffeinated coffee; hot chocolate; ½ milk in coffee or tea; ½ cappuccino or coffee latte.
Alcoholic beverages	White wine, sparkling wine, champagne; red wine; beer; alcoholic aperitifs (vermouth campari, etc); marsala; digestive, sweets liquors, spirits.
Dressing	Oils and animal fats derived from recipes of "vegetables" and "potatoes food groups"

½ indicates recipes or questionnaire items allocated to 2 different food groups.

Figure S2 - Percentage distribution of the different food sources of proteins based on intake in each country

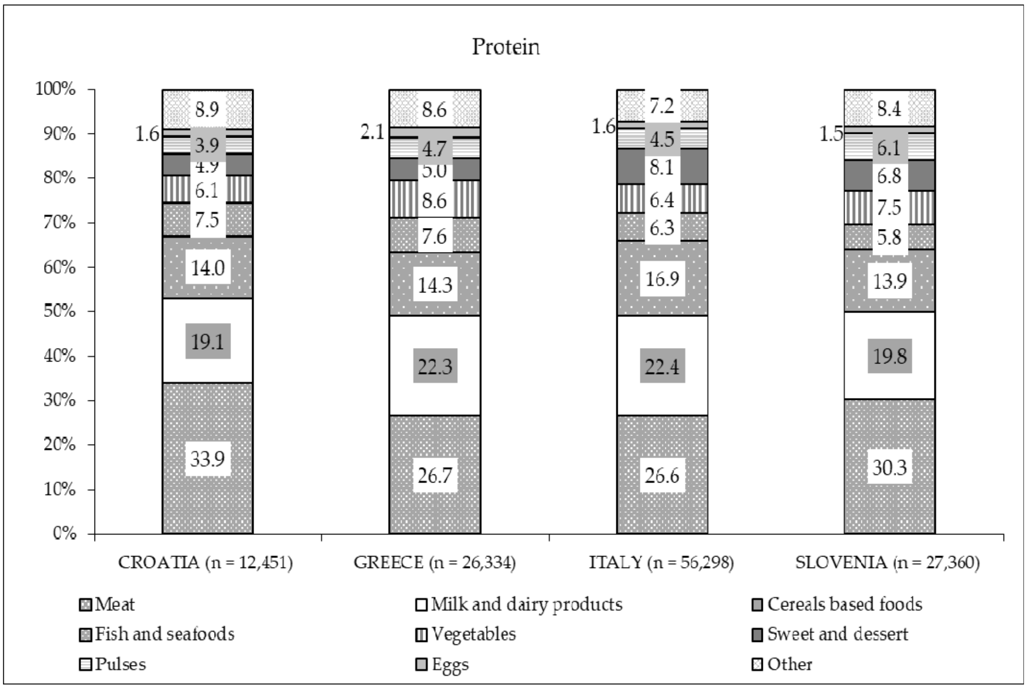

Supplement: Supplementary file 1 [file nutrients-13-01434-s001.zip › nutrients-1144107-supplementary.pdf]
